# Classifying Natural Waters with the Forel-Ule Colour Index System: Results, Applications, Correlations and Crowdsourcing

**DOI:** 10.3390/ijerph121215044

**Published:** 2015-12-18

**Authors:** Shungudzemwoyo P. Garaba, Anna Friedrichs, Daniela Voß, Oliver Zielinski

**Affiliations:** 1 Department of Marine Sciences, Avery Point Campus, University of Connecticut, 1080 Shennecosset Road, Groton, CT 06340, USA; 2 Institute for Chemistry and Biology of the Marine Environment-Terramare, Carl von Ossietzky University of Oldenburg, Schleusenstraße 1, 26382 Wilhelmshaven, Germany; anna.friedrichs@uni-oldenburg.de (A.F.); daniela.voss@uni-oldenburg.de (D.V.); oliver.zielinski@uni-oldenburg.de (O.Z.)

**Keywords:** apparent colour of water, remote sensing, Forel-Ule index, citizen science, water quality

## Abstract

Societal awareness of changes in the environment and climate has grown rapidly, and there is a need to engage citizens in gathering relevant scientific information to monitor environmental changes due to recognition that citizens are a potential source of critical information. The apparent colour of natural waters is one aspect of our aquatic environment that is easy to detect and an essential complementary optical water quality indicator. Here we present the results and explore the utility of the Forel-Ule colour index (FUI) scale as a proxy for different properties of natural waters. A FUI scale is used to distinguish the apparent colours of different natural surface water masses. Correlation analysis was completed in an effort to determine the constituents of natural waters related to FUI. Strong correlations with turbidity, Secchi-disk depth, and coloured dissolved organic material suggest the FUI is a good indicator of changes related to other constituents of water. The increase in the number of tools capable of determining the FUI colours, (i) ocean colour remote sensing products; (ii) a handheld scale; and (iii) a mobile device app, make it a versatile relative measure of water quality. It has the potential to provide higher spatial and temporal resolution of data for a modernized classification of optical water quality. This FUI colour system has been favoured by several scientists in the last century because it is affordable and easy to use and provides indicative information about the colour of water and the water constituents producing that colour. It is therefore within the scope of a growing interest in the application and usefulness of basic measurement methodologies with the potential to provide timely benchmark information about the environment to the public, scientists and policymakers.

## 1. Introduction

The need for fast, inexpensive, simple, automated, and non-invasive technology in operational aquatic environmental monitoring has grown in recent decades. Measurements and observations taken with such tools provide essential information with respect to bio-geophysical water quality [1,2,3,4,5]. Here we present the Forel-Ule colour index (FUI) system, a colorimetric approach that has a longstanding history [2,6,7] and a standard output of the widely used radiative transfer numerical model, the Sequoia Scientific Hydrolight. The FUI system is practical, assigning one numerical value on a colour scale ranging from 1 (indigo-blue) to 21 (cola brown), and can be derived from ocean colour remote sensing products, including remote sensing reflectance (*R_RS_*) or water leaving radiance [1,6,8]. The benefit of using *R_RS_* is that it has been classified as an essential climate variable vital in inferring optically active agents of water [9,10]. A concise background and history of the FUI system is well-documented in open access literature [6,11].

Natural waters absorb and scatter light in the presence of colour producing agents (CPAs), thereby determining the apparent colour of surface water masses. Primary CPAs include coloured dissolved organic matter (CDOM, also called Gelbstoff), inorganic suspended particulate material (SPM or mineral solids) and particulate organic matter (POM or phytoplankton chlorophyll-*a* (chl-*a*), the most abundant pigment) [2,12]. It is important to note that while there are other pigments that do absorb light in the ocean, chl-*a* is the most abundant pigment which is widely used to represent POM or biomass. Therefore, the apparent colour of water based on any colorimetric scale like the FUI colour system is influenced by the extent of inherent additive absorption and scattering of CPAs. To this end, various localized studies throughout the global oceans have recognized and confirmed that apparent colour of water or optical measurements are proportional to a number of water quality variables [1,2,5,8,13,14,15,16,17].

Typical water quality variables that are correlated to FUI include turbidity and Secchi-disk depth (SDD). Turbidity is a relative measure of water haziness resulting from inherent dissolved and suspended particulate material [18,19]. SDD is a simple and inexpensive traditional index of average vertical visual water transparency or aquatic light penetration depth [20,21]. SDD is a common proxy of the trophic status of a water mass that is related to the apparent colour of water, concentrations of CPAs and environmental perturbations [2,8,16,22].

Knowledge of water quality status with respect to light is a valuable tool in understanding changes in the aquatic environment—for example, the role of light in ecosystem health, aesthetics, and primary production. In this work, we concentrate on the FUI colour system, which provides a low-cost and easy-to-use approach of monitoring the marine environment [11]. We showcase the results and usefulness of the FUI in (i) mapping apparent colour of water changes over large temporal and areal expanses; (ii) monitoring time series colour of water changes in an estuarine system; (iii) evaluating if colour can be derived from CPAs only; and (iv) assessing its possible use as a water quality proxy that can be used in predicting other optical water quality variables such as turbidity and SDD. In this study, the correlations investigated were based on *in situ* observations from several water bodies. It therefore improves and supports prior knowledge on how FUI colours are related to CPAs as previously reported using simulated data [17]. Advances in technology have seen an increase in the number of tools that are used to determine the FUI. It was typically observed using a handheld colour scale, but can now be derived from ocean colour remote sensing products and via a smartphone app, an indication that it has a promising future in operational optical observations of the aquatic environment.

## 2. Methods and Sampling

Field sampling was carried out in the northwestern European shelf seas between April and May 2009, the Faroe Islands, Skagerrak strait, Baltic Sea and North Sea between May and June 2011, off the west coast of Greenland and Iceland between July and August 2012, the Elbe estuarine system in May 2012 and May 2013, Wadden Sea time series station Spiekeroog between August and October 2013, and the Norwegian Seas and Skagerrak strait between August and September 2014. Measurements used here are available on request via email and by open access via the PANGAEA repository [23,24,25,26,27,28,29,30].

### 2.1. Derivation of FUI from Ocean Colour Remote Sensing Products

The remote sensing reflectance or water-leaving radiance (both ocean colour remote sensing products) are first corrected for surface-reflected glint [1,31]. The respective ocean colour remote sensing product spectrum is changed into colour by convolution with 1931 CIE–20–Colour Matching Functions to obtain tristimuli values. These tristimuli values are related to the three primary colours: red, green, and blue. The values obtained are then mapped onto a chromaticity diagram and matched to the 21 discrete numbers of the FUI scale. A full description of the algorithm and background on the FUI is available in several prior studies [6,11,32,33]; the MATLAB code is available upon request.

### 2.2. Handheld FUI Scale

**Figure 1:**
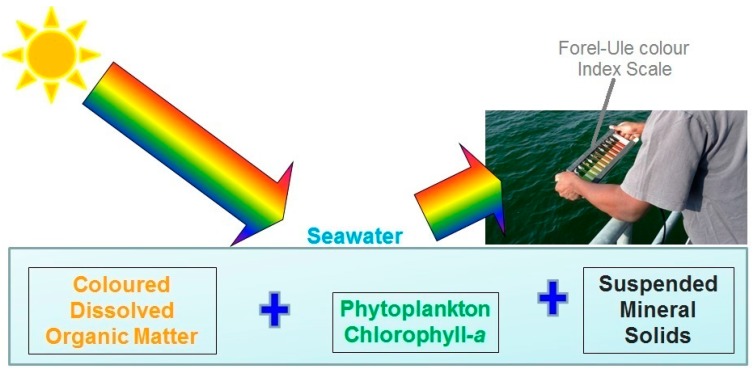
A schematic of the colour producing agents of water and a user observing the optical colour of seawater using a handheld Forel-Ule colour index scale. The Secchi-disk is not visible in the schematic but is held at half Secchi-disk depth.

It is recommended that one first determine the SDD and then raise the Secchi-disk to half that value. Whilst it is at half SDD, the best matching colour of the water mass is identified by looking at the colour tubes or strips of the handheld FUI colour scale over the water mass surface immediately over the submerged Secchi-disk [32]. A simplified overview (Figure 1) of a handheld scale in the field illustrates the CPAs that contribute to the observed colours and path of sunlight. To mitigate environmental contamination of the observations it is highly recommended to avoid surface-reflected glitter by positioning the user’s back to the sun and standing in the shade if possible [32].

### 2.3. CPAs and Water Clarity

Visual water clarity was measured using a 20–40 cm diameter Secchi-disk subject to environmental conditions. Turner Designs CYCLOPS-7, Seapoint Sensors and WETlabs Eco FLNTU sensor turbidity meters were used to measure the relative water cloudiness or turbidity [NTU]. CDOM absorption coefficient at 440 nm (m^−1^) was computed from the absorption coefficient at 275 nm (m^−1^) and the spectral slope in the wavelength range 275 nm to 295 nm from a non–linear least squares exponential fit. SPM (mg/L) was measured after gravimetric and combustion procedures. Chl-*a* [μg/L] was determined according to the United States Environmental Protection Agency Method 445.0 [34]. A detailed description of these steps is described in a recent study [8]. All the CDOM, SPM and chl-*a* measurements utilized in this study were collected *in situ*.

### 2.4. Statistical Analysis

In order to quantify differences and uncertainties in the predictive regression equations, we computed mean absolute percent difference in Equation (1), which represents how the data is scattered; the mean percent difference in Equation (2) is an indicator of bias.

(1) $$\psi_{mapd}=100\times \frac{1}{N}\sum_{i=1}^{N}\frac{\left|X_{predicted}-X_{in-situ}\right|}{X_{in-situ}}$$

(2) $$\psi_{mpd}=100\times \frac{1}{N}\sum_{i=1}^{N}\frac{X_{predicted}-X_{in-situ}}{X_{in-situ}}$$

where *N* is the number of available observations and *X* is the variable observed. Furthermore, the degree of association among the measured variables was determined using a Spearman rank correlation test in MathWorks Matlab 2014b. A Spearman test was applied because it permits non-parametric tests for monotonic associations.

## 3. Results

### 3.1. Mapping Apparent Colours of Water

**Figure 2:**
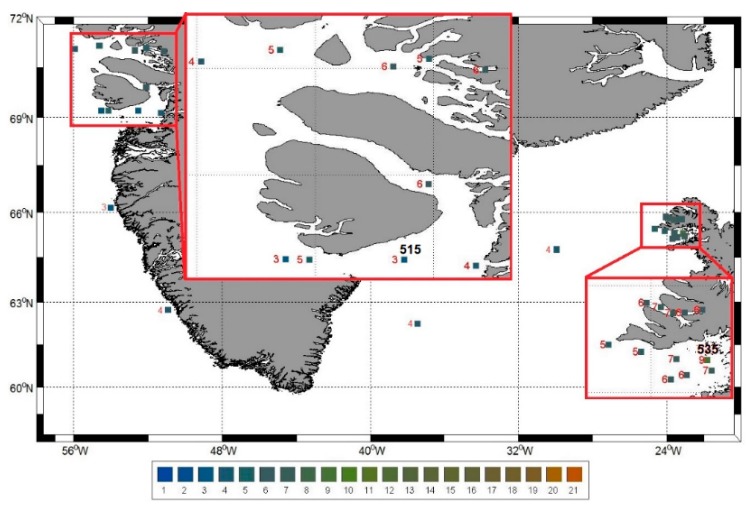
A FUI colour map of the visual observations from the west of Greenland and Iceland aboard R/V *Maria S. Merian* between 27 July and 8 August 2012.

A standard handheld FUI colour scale was used at 26 stations off the west of Greenland and Iceland to determine the apparent colour of the water and a representative map (Figure 2) of the observed FUI colours was produced. We related the FUI values with matching water quality variables and it was noted that, for example, (i) station 535 had FUI = 9, greenish waters, which had relatively high chl-*a* = 7.5 μg/L, low SDD = 5 m, and high turbidity = 3.6 NTU; (ii) station 515 had FUI = 3, bluish waters, with relatively low chl-*a* = 0.2 μg/L, high SDD = 13.5 m, and low turbidity = 0.2 NTU.

**Figure 3:**
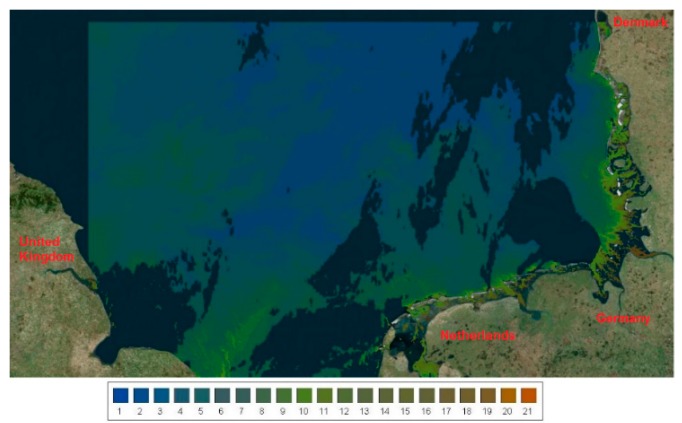
FUI colours derived from MERIS full-resolution imagery captured on 2 May 2009. Image was generated using the online marine data analyser [36].

Mapping the colour of water over a large area is instrumental in tracking both small-scale and large-scale dynamics in the surface waters. Recently, selected medium resolution imaging spectrometer (MERIS) satellite imagery was analysed and converted into FUI colour maps using the FUME algorithm [35]. Using an image captured on 2 May 2009, we provide a FUI colour map of the North Sea (Figure 3). Similar images are available through the European-Commission-funded project CITCLOPS (Citizens’ Observatory for Coast and Ocean Optical Monitoring) homepage [36].

**Figure 4:**
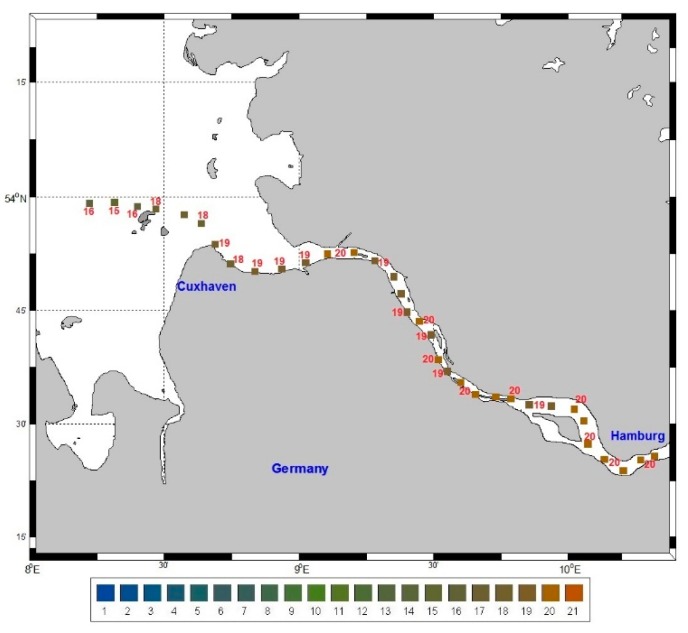
FUI colours of water derived from remote sensing reflectance measured in the Elbe estuarine system in May 2013. The FUI colours were validated and corrected using field observations with a handheld FUI colour scale.

FUI observations using a handheld colour scale are instrumental in validating and correcting the FUI derived from ocean colour remote sensing products like *R_RS_* or smartphone apps. A field campaign was carried out in the Elbe estuarine system, (Figure 4) in which the apparent colour of the water was recorded using a handheld FUI colour scale at 25 stations. To complement these FUI observations, *R_RS_* was also determined at 34 stations. The two datasets of FUI determined from a handheld colour scale (*i.e.*, our reference measurement) and *R_RS_* were assessed for uncertainties. It was noted that FUI values derived from *R_RS_* had relatively low uncertainties, with a mean bias and scatter of 6% ± 5.5%. A linear fit was applied to determine a correction approach so that we could map the FUI at 34 stations derived from *R_RS_* instead of only at 25 stations determined by the handheld colour scale. A map (Figure 4) was created using these validated and corrected FUI colours. A similar approach that involved producing a plausible FUI map from measured *R_RS_*, because a handheld colour scale was not available, was recently presented in a prior study [8].

**Figure 5:**
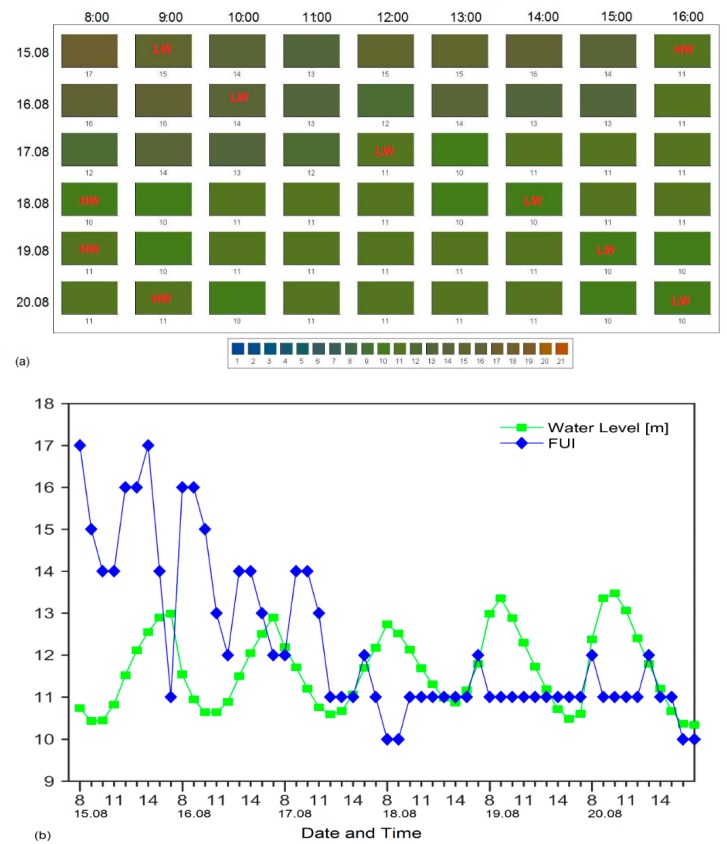
(**a**) Hourly averaged changes in Forel-Ule Index (FUI) colour of water and (**b**) hourly averaged water level and FUI colour changes between 08:00 and 16:00 UTC, from 15 August to 20 August 2013 at the Wadden Sea time series station Spiekeroog. HW indicates high water level and LW indicates low water level.

In order to further promote the utility of the FUI system, we illustrate its use by looking at averaged hourly apparent colour changes over a few days and tidal phases (Figure 5) at a fixed platform located at a tidal inlet of the Wadden Sea (53°45.01644′ N, 7°40.26552′ E). Near water surface *R_RS_* information from the Wadden Sea time series station Spiekeroog was used to infer FUI colours around the platform [1]. Ocean colour remote sensing at the station depends on sunlight and, therefore, valid measurements were limited to a time interval between 8:00 and 16:00 UTC (*i.e.*, corresponding to hours of optimum daylight).

### 3.2. FUI as a Proxy for Water Clarity Parameters

**Figure 6:**
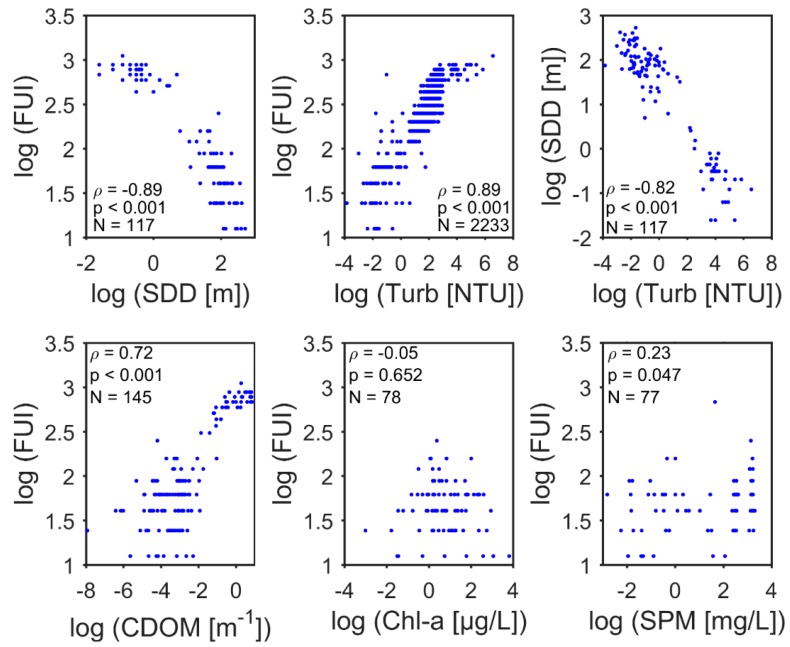
Spearman’s rank correlation test statistics from observed Forel-Ule Index (FUI), Secchi-disk depth (SDD), turbidity (Turb), coloured dissolved organic matter (CDOM), chlorophyll-*a* (chl-*a*), and inorganic suspended particulate material (SPM). The statistics are correlation coefficients (ρ), number of points (N) and a statistical significance *p*-value.

SDD and turbidity are common water transparency indicators that provide some information about the penetration depth of light in an aquatic environment. As an increasing number of studies have shown, these indicators are associated with FUI [1,2,8,16]. Here, we confirm the relationship between apparent colour of water based on the FUI colour scale with both SDD and turbidity. We use the Spearman rank correlation test, which shows very strong correlations (Figure 6).

FUI colour scale values were observed to decrease with decreasing turbidity (Figure 6). This is consistent with the theory that as water becomes more turbid or muddy (turbidity increases) with increasing suspended particulate and dissolved material, the apparent colour darkens (higher FUI colour values—brownish waters). The light penetration depth as determined with a SDD will be low. Thus, SDD is indirectly correlated with both turbidity and FUI (Figure 6). In general, low turbidity corresponds to clear blue waters (*i.e.*, low FUI and high SDD). Low SDD corresponds to turbid waters with very high FUI. The correlation test (Figure 6) supports the utility of an inexpensive and simple technique, the FUI scale, as a proxy of optical water quality or trophic level state.

### 3.3. Inferring the FUI from CPAs

It is widely known that the apparent colour of water is a product of additive inherent optical properties of CPAs, environmental conditions and human eye perception. Simulations using the Hydrolight radiative transfer numerical model have shown that the apparent colour of water, as measured by the FUI colour system, is correlated to the concentrations of the three primary CPAs [17]. Taking advantage of *in situ* observations of these primary CPAs (*i.e.*, CDOM, chl-*a* and SPM), we assessed the degree of correlations with observed *in situ* or derived FUI. Spearman rank correlation tests with log-transformed (Figure 6) field observations confirmed some of the theoretical results; FUI had a strong correlation with CDOM, very weak with chl-*a*, and weak with SPM (Figure 6). Additionally, multivariate regression analysis was completed (Table 1). CDOM was a primary driver of colour for these near shore waters and is supported by its significant strong correlation with FUI (Figure 6). As the retrieval uncertainties were considerable, we believe this relationship merits further investigation and can be useful in most qualitative analyses. Our assumption that only the CPAs can be used to derive the FUI colour of water is plausible with caveats, as we believe for quantitative analysis future studies have to account for the environmental perturbations and limitations of such a relationship. The west Greenland and Iceland regression was statistically significant (*p* < 0.05), whilst that for the northwestern European Seas was statistically significant (*p* < 0.01).

**Table 1:** Multivariate linear regressions linking Forel–Ule colour Index (FUI) to coloured dissolved organic matter (CDOM), chlorophyll-*a* (chl-*a*), and non-algal particles (SPM). Regression statistics are number of available matching points (*N*), regression coefficient (*R²*), mean absolute percent difference (*ψ_mapd_*), and mean percent difference (*ψ_mpd_*).

Region	*FUI =*	*N*	*R^2^*	$\psi_{mapd}$ **	$\psi_{mpd}$ **
North-western European Seas	4.4 + 19.4 × CDOM − 0.1 × chl-*a* + 0.06 × SPM	47	0.25 ^a^	21 ± 16	6 ± 26
West Greenland, Iceland	4.6 + 9.5 × CDOM + 0.3 × chl-*a* − 0.05 × SPM	24	0.32 ^b^	22 ± 16	6 ± 27

^a^, *p* < 0.01; ^b^, *p* < 0.05.

## 4. Discussion

### 4.1. Mapping Colour of Surface Waters

The need to document the colour of natural waters can be traced back to the works of François Alphonse Forel and Willi Ule in the 1890s, hence the development of the FUI colour scale [6]. This scale has been favoured by several scientists in the last century because it is affordable, easy to use and provides meaningful information about colour of water as well as water constituents driving colour (e.g., [1,2,8,16,17]). In this study we used the FUI colour scale and showcase results from field campaigns that aimed at distinguishing natural water masses based on the apparent colour of water as observed using the FUI colour scale. Equipped with additional matching water quality variables, we highlight how FUI can be a useful qualitative proxy correlated with other constituents of natural waters. A link between colour and water constituents was noted, but a sensitivity analysis of these correlations will contribute to our understanding of the ocean and colour-related dynamics or changes. A sensitivity analysis will require vast amounts of accurate and extensive measurements of CPAs, inherent optical properties, turbidity and SDD to match FUI colour observations. Matching *in situ* data is very limited; for example, in a recently compiled dataset from 1890 to 2010 [37] there are records of FUI colour observations and other water constituents, but very few of these compiled data match.

Ocean colour remote sensing products have become key parameters in understanding the environment especially over long time scales and large areas. *R_RS_* is the key product which can be translated into concentrations of CPAs utilising bio-optical algorithms [38] or colour of water based on the FUI colour system using international colour standards [6]. FUI colours derived from collected *R_RS_* were calibrated and validated using field observations with a handheld scale. Here we show the utility of ocean colour remote sensing in reducing gaps during field campaigns (Figure 4). Instead of having only 25 stations where a handheld scale was used to observe FUI, we ended up with 34 stations from the FUI colour information derived from *R_RS_*. Furthermore, we demonstrate that using satellite imagery (Figure 3) we can determine the colour of water based on the FUI system, which is useful in large scale detection and time series tracking of surface water plumes or even algal blooms. It is therefore anticipated that future sea truth observations of FUI colours matching multispectral to hyperspectral airborne or satellite overpass will be capable of providing a large spatial coverage of observations on the colour of the sea.

### 4.2. Monitoring Changes in Colour of Water

Scientists, policy makers and citizens rely heavily on timely warnings based on operational and continuous monitoring of proxies or actual measurements of environmental properties. Colour of water is a classic relative indicator in water that has been instrumental in detecting harmful algal blooms, release of contaminants and track plumes [4,39]. Observations of colour can be collected using near water surface, airborne and satellite platforms. Each platform has its benefits and drawbacks in terms of areal and temporal resolution, as discussed in previous works [5,39]. Operational fixed platforms like the Wadden Sea time series station Spiekeroog have the capability to produce FUI colour changes up to every minute (near real-time) or averaged weekly changes (Figure 5). Such a platform can serve as an early warning station for rapid changes in natural waters that can be related to other water constituents such as indicators of eutrophication (*i.e.*, turbidity, fluorescence, phosphorus and dissolved oxygen). A recent example would be the recent wastewater spill from the Gold King Mine into the Anima River in Colorado, USA where a rapid change in the colour of river water from relatively clear to yellowish-orange was observed. Here, without operational continuous measurement of biogeochemical properties of the river, the best inexpensive and non-invasive indicator of environmental change would have been the apparent colour. Unfortunately, sensors on fixed platforms near the water’s surface, despite high spectral and temporal resolution, fall short of the areal coverage needed for tracking or detecting changes over large areas. Airborne or satellite sensors are therefore complementary tools with large spatial coverage.

As a new generation of satellite missions (e.g., the Ocean and Land Colour Instrument from the European Space Agency and the Pre-Aerosol, Clouds, and Ocean Ecosystem mission from the National Aeronautics and Space Administration) become fully operational in the next decade, the derivation of FUI colour from satellite remote sensing will greatly improve as these new sensors offer more wavebands in the visible spectrum necessary to accurately convert ocean colour remote sensing products into FUI colours or hue angles [40]. Additionally, missions with short revisit cycles are potential sources of crucial information (temporal dynamics) and are therefore beneficial to operational environmental monitoring. The possibility of deriving FUI from ocean colour remote sensing products (e.g., remote sensing reflectance, water leaving radiance) and a smartphone app makes it a powerful tool for upcoming operational environmental monitoring of water quality for citizens, scientists and policy makers.

### 4.3. Correlations, Relevance and Limitations of FUI as A Proxy

Bivariate correlations (Figure 6 and Section 3.3) were investigated using parameters or variables that are typical measures of water quality that also contribute to apparent colour of water. Here we used *in situ* measurements because several of these correlations have been demonstrated using simulated datasets and the radiative transfer model Hydrolight [17]. We believe the *in situ* data sets here, as well as those from future works, will be key in developing regional to global scale regression models or even semi-analytical models. Another major benefit of *in situ* measurements is that simulations can be evaluated, allowing scientists to determine realistic limitations and threshold of correlations among water quality proxies. It is clear from this study that we are still a long way from establishing universal models, but these empirical models are a starting point for future investigations, especially work towards inexpensive and fast ways to collect information about our environment (e.g., SDD and FUI). Sensitivity analysis would require extensive and accurate *in situ* observations because gaps in matching data for variables linked to apparent colour of water already exist in online repositories. A change in field observation steps is a possible source of uncertainty; for example, personnel can influence FUI observations as colour perception by the human eye is very subjective. Similarly, such uncertainties can be expected for SDD observations as these are visual observations that require consistent measuring steps.

SDD has been shown to be a robust variable useful in deriving chl-*a* at a local to global scale [22,41,42,43]. To this end, FUI recorded since 1890s can be used to retrace spatial and temporal changes based on colour that can be easily associated to concentrations of CPAs, environmental conditions and other water quality variables. Already FUI has been used to successfully retrace chl-*a* at global scale for the period 1890 to 2000 [17]. Time series trends and distributions of retraced chl-*a* based on SDD and FUI can be compared within the framework of tuning ecosystem models. FUI is therefore an essential complementary index of water quality.

Tidal changes involve resuspension of CPAs resulting in colour changes in the water. The changes in apparent colour of water will depend on a number of factors such as variability in concentrations of CPAs, rate at which CPAs are re-suspended, depleted or enhanced, and environmental conditions. In our case here there is no clear correlation between water level and FUI colours (Figure 5); on 15 August 2013 and 15 August 2013, high water periods had light green water FUI = 9 and FUI = 11 whilst the low water periods had dark waters FUI = 16 and FUI = 15, respectively, but this is not the case for other dates from 18 to 20 August 2013, in which FUI colours were relatively constant over the tidal cycle. Ongoing work is exploring this relationship between tidal cycle and colour of water based on FUI.

Using multivariate regression analysis we show that *in situ* CDOM, chl-*a* and SPM can be used to predict the colour of water as measured by a FUI colour scale (Table 1). The low correlation coefficients and the statistical significances are proof that our regression models are not by chance. However, these models also reveal some facts, despite subjective human eye colour perception; (i) CPAs can only provide qualitative and semi-quantitative information related to FUI colour changes; (ii) the FUI cannot necessarily be used to predict individual CPAs; (iii) the relationship between CPAs and FUI based on our dataset is local and (iv) we are still missing some parts of the puzzle. These pieces include knowledge and understanding of other measurable environmental variables that contribute to apparent FUI colours such as wind speed, cloud cover, location and accuracy of measurements.

The FUI scale is limited to discrete numerical values, 1–21, and therefore correlations with other continuous constituents of water will be affected by clustering as previously reported [1]. The clustering effect in the previous study [1] was eliminated by adjusting the *R_RS_* derived FUI indices from discrete to continuous one-decimal-place numerical values; the MATLAB code is available upon request. Alternatively, the hue angle, a continuous indicator of colour of water [40], can be used to mitigate the challenges of correlating the classic discrete FUI indices to other continuous water constituents like turbidity, SDD or CPAs. Applying the hue angle is however restricted to the availability of matching *in situ* FUI observations for validation purposes and ocean colour remote sensing data with enough spectral bands to accurately resolve colours related to the FUI scale. Certainly, the FUI colour system is subjective as much as determining an exact SDD [2,44], but there is a huge amount of *in situ* FUI recordings since the 1890s to date. The strength of the FUI, therefore, lies in its usefulness as a long-term series proxy for changes related to colour in natural waters.

## 5. Conclusions and Future Directions

In this study we evaluated the potential utility of the FUI colour system as a proxy for the classification of water masses, taking advantage of its associations with typical water quality variables. The FUI colour system, as a low-cost and easy-to-use tool, is a robust optical water quality proxy applicable for monitoring the marine environment even when its ability to identify which CPAs are responsible for each colour index is still not fully understood. Field investigations to explore its use were carried out at sea and in estuarine systems in mid-to-high-latitude regions. Correlation and regression analyses were used to show that FUI was well-associated with several optical water quality variables. FUI was positively correlated with turbidity while it was negatively correlated with SDD. It was also shown, with caveats, that the apparent colour of water as observed by the FUI colour system was driven by the concentration (and composition) of the primary CPAs (*i.e.*, CDOM, chl-*a* and SPM). The possibility of deriving FUI from ocean colour remote sensing products (e.g., remote sensing reflectance and water leaving radiance) and a smartphone app makes it a powerful tool for upcoming operational environmental monitoring of water quality by both citizens and scientists. The reasonable accuracy in FUI values from the different sensors provides a form of precision-accuracy validation, a crucial step in ocean colour studies. Furthermore, as monitoring tools accumulate vast amounts of information, there will be a constant need to validate and verify the quality of the information in future works.

**Figure 7:**
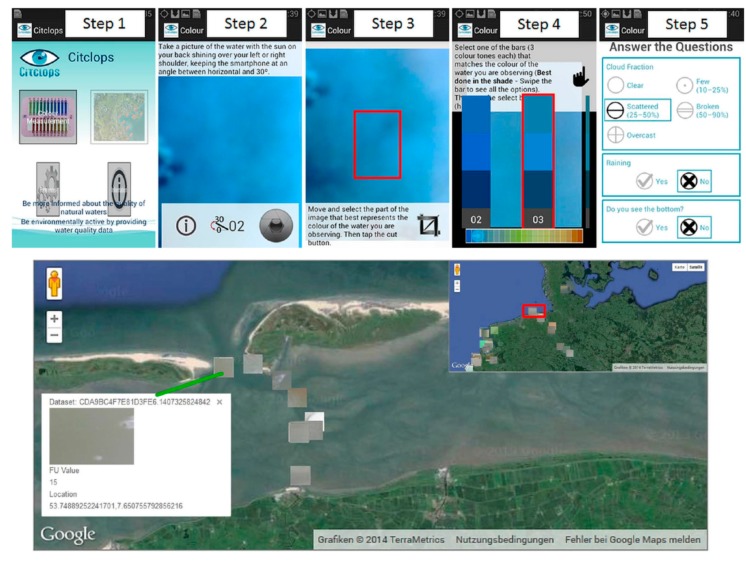
Screenshots of the smartphone app in use over an artificial water surface. Sampling steps: (**1**) open the app and select “Colour measurements”; (**2**) take a picture of the water surface within the given angle range and select the camera shutter icon; (**3**) crop out the part of the images that best represents the apparent colour of the local water surface; (**4**) match the cropped out image to the colour bar and (**5**) complete the short questionnaire and send the data.

The growing environmental awareness by citizens is displayed in a number of ongoing projects. Within the CITCLOPS project [36], a smartphone app is being developed to observe the optical colour of water based on the FUI system. The goal is to benefit from more and more citizens owning smartphones, making it easy and convenient to replace the FUI handheld scale with the smartphone app shown in Figure 7. Algorithms implemented in the app are well-established and continue to be updated based on previous and ongoing studies [6,33,45]. The determination and recording of the apparent colour of water can be performed using a smartphone app in five easy-to-handle steps (Figure 7). Within the app, other optical water quality variables, such as SDD and handheld FUI observations, can be recorded as auxiliary information along with further metadata. A central database [36] is used for storage of the evaluated data in order to generate an open-access water colour map based on the FUI system. The image and calculated FUI will be displayed on the map matching the evaluation criteria (Figure 7). In the near future, additional variables such as fluorescence and transparency will be integrated into the map. Images sent to the database are now processed using the new water colour from digital images algorithm [33]. With this app and the awareness of citizens participating in environmental monitoring observations helping to understand environmental processes, large data sets can be gained to support scientific studies.

This increasing demand for high-resolution spatial and temporal environmental monitoring echoes the need for new and easy-to-use methods for the collecting of reliable and accurate data sets for water quality control. Examples of ongoing efforts using smartphone apps to determine optical water quality variables include the HydroColor app developed by Thomas Leeuw and Emmanuel Boss at the University of Maine, USA and the Secchi app developed by Nicholas Outram and Nigel Barlow at Plymouth University, UK [46]. Therefore, ocean colour remote sensing, complemented by its predictive algorithms and smartphone apps, has the potential to meet the requirements of future investigations targeting water quality changes.

## References

[1] Garaba S.P., Badewien T.H., Braun A., Schulz A.-C., Zielinski O. (2014). Using ocean colour products to estimate turbidity at The Wadden Sea time series station Spiekeroog. J. Eur. Opt. Soc. Rapid Publ..

[2] Graham J.J. (1966). Secchi disc observations and extinction coefficients in the central and eastern North Pacific Ocean. Limnol. Oceanogr..

[3] Monahan E.C., Pybus M.J. (1978). Colour, ultraviolet absorbance and salinity of the surface waters off the west coast of ireland. Nature.

[4] Platt T., Hoepffner N., Stuart V., Brown C., IOCCG (2008). Why Ocean Colour? The Societal Benefits of Ocean-Colour Technology.

[5] Garaba S.P., Zielinski O. (2015). An assessment of water quality monitoring tools in an estuarine system. Remote Sens. Appl. Soc. Environ..

[6] Wernand M.R. (2011). Poseidons paintbox: Historical archives of ocean colour in global-change perspective. Ph.D. Thesis.

[7] Tyler J.E. (1964). Colour of the ocean. Nature.

[8] Garaba S.P., Voß D., Zielinski O. (2014). Physical, bio-optical state and correlations in north-western European shelf seas. Remote Sens..

[9] Garaba S.P., Zielinski O. (2013). Comparison of remote sensing reflectance from above-water and in-water measurements west of Greenland, Labrador sea, Denmark strait, and west of Iceland. Opt. Express.

[10] GCOS (2011). The Global Climate Observing System-Systematic Observation Requirements for Satellite-Based Data Products for Climate: 2011 Update GCOS-154.

[11] Novoa S., Wernand M.R., van der Woerd H.J. (2014). The modern Forel-Ule scale: A “do-it-yourself” colour comparator for water monitoring. J. Eur. Opt. Soc. Rapid Publ..

[12] Prieur L., Sathyendranath S. (1981). An optical classification of coastal and oceanic waters based on the specific spectral absorption curves of phytoplankton pigments, dissolved organic matter, and other particulate materials. Limnol. Oceanogr..

[13] Garaba S.P., Zielinski O. (2014). Bio-optical and physical state of high latitude surface waters around south Greenland. Ocean Optics Conference XXII 2014.

[14] Koenings J.P., Edmundson J.A. (1991). Secchi disk and photometer estimates of light regimes in Alaskan lakes: Effects of yellow color and turbidity. Limnol. Oceanogr..

[15] Visser M.P. (1967). Secchi disch and sea colour observations in the North Atlantic Ocean during the navado III cruise, 1964–1965, Aboard H. Neth. M.S. “Snellius” (royal Netherlands navy). Neth. J. Sea Res..

[16] Sakuno Y. (2012). Relationship between water color and transparency in the closed water area based on the chromaticity theory. J. Jpn. Soc. Civ. Eng..

[17] Wernand M.R., van der Woerd H.J., Gieskes W.W.C. (2013). Trends in ocean colour and chlorophyll concentration from 1889 to 2000, worldwide. PLoS ONE.

[18] Kirk J.T.O. (1985). Effects of suspensoids (turbidity) on penetration of solar radiation in aquatic ecosystems. Hydrobiologia.

[19] Andersen C.W. Turbidity (Version 2.1, 9/2005). http://pubs.water.usgs.gov/twri9A6/.

[20] Tyler J.E. (1968). The secchi disc. Limnol. Oceanogr..

[21] Megard R.O., Settles J.C., Boyer H.A., Combs W.S. (1980). Light, secchi disks, and trophic states. Limnol. Oceanogr..

[22] Carlson R.E. (1977). A trophic state index for lakes. Limnol. Oceanogr..

[23] Voß D., Garaba S., Henkel R., Krock B., Zielinski O. Coloured Dissolved Organic Material (CDOM) Absorption Measurements from Samples Collected during The North Sea Coast Harmful Algal Bloom (NORCOHAB II) RV HEINCKE Cruise HE302. PANGAEA—Data Publisher for Earth & Environmental Science, 2010. http://doi.pangaea.de/10.1594/PANGAEA.754378.

[24] Garaba S., Henkel R., Krock B., Voß D., Zielinski O. Forel-Ule Indices from Optical Measurements Collected during The North Sea Coast Harmful Algal Bloom (NORCOHAB II) RV HEINCKE cruise HE302. PANGAEA—Data Publisher for Earth & Environmental Science, 2011. http://doi.pangaea.de/10.1594/PANGAEA.757080.

[25] Zielinski O., Voß D., Meier D., Henkel R., Holinde L., Garaba S.P., Cembella A. Colored Dissolved Organic Matter during Maria S Merian Cruise MSM21/3 (ARCHEMHAB). PANGAEA—Data Publisher for Earth & Environmental Science, 2013. http://doi.pangaea.de/10.1594/PANGAEA.810861.

[26] Zielinski O., Voß D., Meier D., Henkel R., Holinde L., Garaba S.P., Cembella A. Water Transparency Measurements with Secchi Disk during Maria S. Merian Cruise MSM21/3 (ARCHEMHAB). PANGAEA—Data Publisher for Earth & Environmental Science, 2013. http://doi.pangaea.de/10.1594/PANGAEA.810648.

[27] Zielinski O., Voß D., Meier D., Henkel R., Holinde L., Garaba S.P., Cembella A. Physical oceanography during Maria S. Merian cruise MSM21/3 (ARCHEMHAB). PANGAEA—Data Publisher for Earth & Environmental Science, 2013. http://doi.pangaea.de/10.1594/PANGAEA.819731.

[28] Zielinski O., Voß D., Meier D., Henkel R., Holinde L., Garaba S.P., Cembella A. Forel-Ule Indices Observed during Maria S. Merian Cruise MSM21/3 (ARCHEMHAB). PANGAEA—Data Publisher for Earth & Environmental Science, 2013. http://doi.pangaea.de/10.1594/PANGAEA.810707.

[29] Zielinski O., Voß D., Meier D., Henkel R., Holinde L., Garaba S.P., Cembella A. Chlorophyll A during Maria S. Merian Cruise MSM21/3 (ARCHEMHAB) PANGAEA—Data Publisher for Earth & Environmental Science, 2013. http://doi.pangaea.de/10.1594/PANGAEA.810648.

[30] Zielinski O., Voß D., Meier D., Henkel R., Holinde L., Garaba S.P., Cembella A. Total Suspended Matter, Particulate Organic Matter, and Particulate Inorganic Matter during Maria S. Merian Cruise MSM21/3 (ARCHEMHAB). PANGAEA—Data Publisher for Earth & Environmental Science, 2013. http://doi.pangaea.de/10.1594/PANGAEA.810708.

[31] Garaba S.P., Zielinski O. (2013). Methods in reducing surface reflected glint for shipborne above-water remote sensing. J. Eur. Opt. Soc. Rapid Publ..

[32] Wernand M.R., van der Woerd H.J. (2010). Spectral analysis of the Forel-Ule ocean colour comparator scale. J. Eur. Opt. Soc. Rapid Publ..

[33] Novoa S., Wernand M., van der Woerd H.J.W. (2015). A generic algorithm to derive the intrinsic color of natural waters from digital images. Limnol. Oceanogr. Methods.

[34] Arar E.J., Collins G.B. (1997). Method 445.0: In Vitro Determination of Chlorophyll A and Pheophytin A in Marine and Freshwater Algae by Fluorescence.

[35] Wernand M.R., Hommersom A., van der Woerd H.J. (2013). Meris-based ocean colour classification with the discrete Forel–Ule scale. Ocean Sci..

[36] CITCLOPS Citizens’ Observatory for Coast and Ocean Optical Monitoring. European Commission (Grant Agreement Number fp7–env-2012-308469). http://www.citclops.eu/.

[37] Boyce D.G., Lewis M., Worm B. (2012). Integrating global chlorophyll data from 1890 to 2010. Limnol. Oceanogr. Methods.

[38] Morel A., John H.S. (2001). Bio-optical models. Encyclopedia of Ocean Sciences.

[39] Zielinski O., Busch J.A., Cembella A.D., Daly K.L., Engelbrektsson J., Hannides A.K., Schmidt H. (2009). Detecting marine hazardous substances and organisms: Sensors for pollutants, toxins, and pathogens. Ocean Sci..

[40] Van der Woerd H., Wernand M. (2015). True colour classification of natural waters with medium-spectral resolution satellites: SeaWiFS, MODIS, MERIS and OLCI. Sensors.

[41] Megard R.O., Berman T. (1989). Effects of algae on the secchi transparency of the Southeastern Mediterranean Sea. Limnol. Oceanogr..

[42] Lewis M.R., Kuring N., Yentsch C. (1988). Global patterns of ocean transparency: Implications for the new production of the open ocean. J. Geophys. Res. Oceans.

[43] Falkowski P.G., Wilson C. (1992). Phytoplankton productivity in the North Pacific Ocean since 1900 and implications for absorption of anthropogenic CO_2_. Nature.

[44] Preisendorfer R.W. (1986). Secchi disk science: Visual optics of natural waters. Limnol. Oceanogr..

[45] Novoa S., Wernand M.R., van der Woerd H.J. (2013). The Forel-Ule scale revisited spectrally: Preparation protocol, transmission measurements and chromaticity. J. Eur. Opt. Soc. Rapid Publ..

[46] Carling B. (2013). A new way of measuring phytoplankton. Mar. Sci..

